# Establishment of Human Patient-Derived Endometrial Cancer Xenografts in NOD *scid* Gamma Mice for the Study of Invasion and Metastasis

**DOI:** 10.1371/journal.pone.0116064

**Published:** 2014-12-26

**Authors:** Kenji Unno, Masanori Ono, Abigail D. Winder, Kruti P. Maniar, Ajit S. Paintal, Yanni Yu, Jian-Jun Wei, John R. Lurain, J. Julie Kim

**Affiliations:** 1 Department of Obstetrics and Gynecology, Division of Reproductive Science in Medicine, Northwestern University Feinberg School of Medicine, Chicago, IL, 60611, United States of America; 2 Department of Obstetrics and Gynecology, Division of Gynecologic Oncology, Northwestern University Feinberg School of Medicine, Chicago, IL, 60611, United States of America; 3 Department of Pathology, Northwestern University Feinberg School of Medicine, Chicago, IL, 60611, United States of America; University of Quebec at Trois-Rivieres, Canada

## Abstract

**Objective:**

Most endometrial cancers are detected early and have a good prognosis, while some endometrial cancers are highly invasive, metastasize early, and respond suboptimally to therapy. Currently, appropriate model systems to study the aggressive nature of these tumors are lacking. The objective of this study was to establish a mouse xenograft model of endometrial tumors derived from patients in order to study the biological aggressive characteristics that underlie invasion and metastasis.

**Methods:**

Endometrial tumor tissue fragments (1.5 mm×1.5 mm) from patients undergoing surgery, were transplanted under the renal capsule of NOD *scid* gamma mice. After 6–8 weeks, tumors were excised and serially transplanted into additional mice for propagation. Immunohistochemical analysis of the tumors was done for various tumor markers.

**Results:**

Four cases of different subtypes of endometrial cancer were grown and propagated in mice. Three of the four tumor cases invaded into the kidneys and to adjacent organs. While all tumors exhibited minimal to no staining for estrogen receptor α, progesterone receptor staining was observed for tumor grafts. In addition, levels and localization of E-cadherin, cytokeratin and vimentin varied depending on subtype. Finally, all tumor xenografts stained positively for urokinase plasminogen activator while 3 tumor xenografts, which showed invasive characteristics, stained positively for urokinase plasminogen activator receptor.

**Conclusion:**

Endometrial tumors transplanted under the renal capsule exhibit growth, invasion and local spread. These tumors can be propagated and used to study aggressive endometrial cancer.

## Introduction

Endometrial cancer is the fourth most common malignant tumor among women in the United States, with about 49,500 new cases and 8,200 deaths in 2013 [1]. There are two types of endometrial cancers categorized as Type I and Type II, depending on histological and clinical outcomes. Type I which occurs in approximately 75%–85% of the cases of endometrial cancer is associated with unopposed estrogen action with risk factors including obesity, anovulation, and polycystic ovarian syndrome. Type 1 tumors are adenocarcinomas, also termed endometrioid endometrial cancer (EEC) [2]. EEC is characterized by a variety of genetic alterations, including PTEN, β-catenin, PIK3CA, ARID1A, KRAS and ARID5B mutations [3]. Type II cancers include serous carcinoma, clear cell carcinoma, poorly differentiated, grade 3 endometrioid carcinoma, and carcinosarcoma or malignant mixed mullerian tumor (MMMT). These cancers have poorer prognoses and account for 40% of deaths due to endometrial cancer [4], [5]. Uterine serous carcinoma (USC) is a highly aggressive lesion in which the epithelial morphology is similar to ovarian serous carcinoma [6]. USC tends to spread extensively in vascular spaces and through the myometrium [4]. These tumors commonly spread to the abdomen and lymph nodes [7]. The most remarkable genetic alteration of USC is p53 mutation, which occurs in approximately 90% of cases [3]. The p53 mutation leading to protein overexpression is considered to be an early event of USC carcinogenesis, and MSI or mutation of PTEN are not as prevalent [3].

To date, xenograft studies of endometrial cancer have been limited to subcutaneous and orthotopic (uterine) injections of immortalized cell lines [8]–[12]. Whereas growth of subcutaneous xenografts can be easily monitored in a non-invasive fashion, xenografts in orthotopic or other ectopic sites within the body cavity provide a different microenvironment for tumors to grow, often allowing better growth, survival and vascularization. The kidney of NOD *scid* gamma is a site that has been widely used for tissue grafting because this organ provides high level of blood and lymph flow rates, and a positive interstitial fluid pressure [13]. Cells and tissues from both benign and malignant tissues have been successfully grafted under the renal capsule in these mice. These include human endometrial and leiomyoma tissues [14]–[16], prostate cancer, breast tissues [17], [18], ovarian tumor tissues [19], [20].

Therefore, the objectives of this study were to establish and propagate tumors from primary advanced endometrial cancer under the kidney capsule of NOD *scid* gamma mice and to characterize tumors using various markers for tumor subtype, EMT, steroid receptors, and invasion.

## Materials and Methods

### Tissue collection

Endometrial tumors were obtained from women undergoing hysterectomies at Northwestern Memorial Hospital. Patients provided written consent prior to surgery. This study was approved by the Human Subject Committee of Northwestern University in accordance with U.S. Department of Health Regulations.

### Subrenal grafting of human primary endometrial cancer tissues

All procedures involving animals were approved by Northwestern University's Animal Care and Use Committee (Protocol # 2012–2867). All surgical procedures were performed under anesthesia by intraperitoneal injection of ketamine/xylazine (90/8 mg/kg) and all efforts were made to minimize suffering. Adult female NOD *scid* gamma (NSG) mice (Jackson Laboratory, Bar Harbor, ME), were ovariectomized (OVX) and supplemented with or without 0.36 mg E2 in the form of 90-day release pellets (Innovative Research of America Inc., Sarasota, Fl) which were implanted subcutaneously. Endometrial tumor tissue fragments were obtained from patients post-surgery. Tumors were cut into small fragments (1.5 mm×1.5 mm) and grafted under the renal capsule of adult female NSG mice (Jackson Laboratory, Bar Harbor, ME) as previously described [14]. Two fragments per kidney were grafted on the anterior and caudal sides and both kidneys were used. After 6–8 weeks of grafting, tumors were removed, cut into smaller fragments (1.5 mm×1.5 mm) and then transplanted under the renal capsule of NSG mice for serial propagation. Xenografted tissues were labeled as passage 0 (P0), P1, P2, etc depending on the number of passages from the initial tumor. Tumor tissues were fixed in 10% neutral buffered formalin containing 3.8% formaldehyde (VWR International, West Chester, PA) and subsequently paraffin embedded for histological analysis. Portions of the tumor were also snap frozen in liquid nitrogen or cryopreserved and stored at −80C for additional analysis.

Mice were housed in a barrier facility, that is pathogen free, in cages with environmental enrichment, and fed irradiated rodent Teklad diet. Mice were housed for 14 hours light and 10 hours dark cycle. Mice were given analgesics (meloxicam) for pain management for two days post-surgery and observed on a daily basis for signs of distress such as slowed respiration, failure of grooming and fur ruffling and failure to respond to cage tapping. At the end of 6–8 weeks, mice were euthanized by CO_2_ followed by cervical dislocation.

### Immunohistochemistry

Paraffin-embedded sections were deparaffinized and stained using the Envision DAB HRP kit (Dako) or hematoxylin and eosin (H&E). The following primary antibodies were used for IHC; rabbit polyclonal anti-progesterone receptor 1∶1000 (Dako, Cat#A0098), rabbit monoclonal anti-estrogen receptor α 1∶5000 (Abgent, Cat#AJ1268a), rat monoclonal anti-Ki67 1∶31250 (Dako, Cat#M7249), goat polyclonal anti-CD31 1∶1000 (Santa cruz, Cat#Sc-1506), mouse monoclonal anti-E-cadherin 1∶50 (BD Transduction Laboratories, Cat#610181), mouse monoclonal anti-Pan-cytokeratin (recognizing cytokeratins 4, 5, 6, 8, 10, 13 and 18) 1∶500 (Cell Signaling, Cat#4545), rabbit monoclonal anti-Vimentin 1∶2500 (Abcam, ab92547), rabbit monoclonal anti-p53 1∶160 (Cell Signaling, Cat#2527), rabbit monoclonal anti-PTEN 1∶125 (Cell Signaling, Cat#9188), mouse monoclonal anti-uPA 1∶50 (American Diagnostic GmbH, Cat#ADG3689). For the PTEN antibody, SignalStain Antibody Diluent for primary antibody dilution and SignalStain Boost (HRP, Rabbit) for detection from Cell Signaling were used. UPAR (ATN-617 mouse) antibody 1∶300 was kindly provided by Dr. Andrew Mazar (Northwestern University). After incubation of primary antibodies, slides were rinsed in TBS-T and species-specific (anti-rabbit, anti-mouse and anti-goat) secondary antibody conjugated to a dextran labeled polymer and horseradish peroxidase was applied and stained using DAB solution. Images were captured on a Leica DM5000B Microscope.

### Cryopreservation of xenografted tissues

Tissue fragments of 1.5 mm×1.5 mm size (approximately 10 pieces per tube) were placed in a solution of 10% DMSO and 90% FBS and stored at -80°C. Tumor fragments for EEC4 were thawed at room temperature, washed twice with PBS and immediately grafted under the renal capsule of two OVX mice. Seven weeks after grafting, mice were dissected and tumor growth was analyzed.

## Results

### Establishment of xenografts under the renal capsule

**Figure 1:**
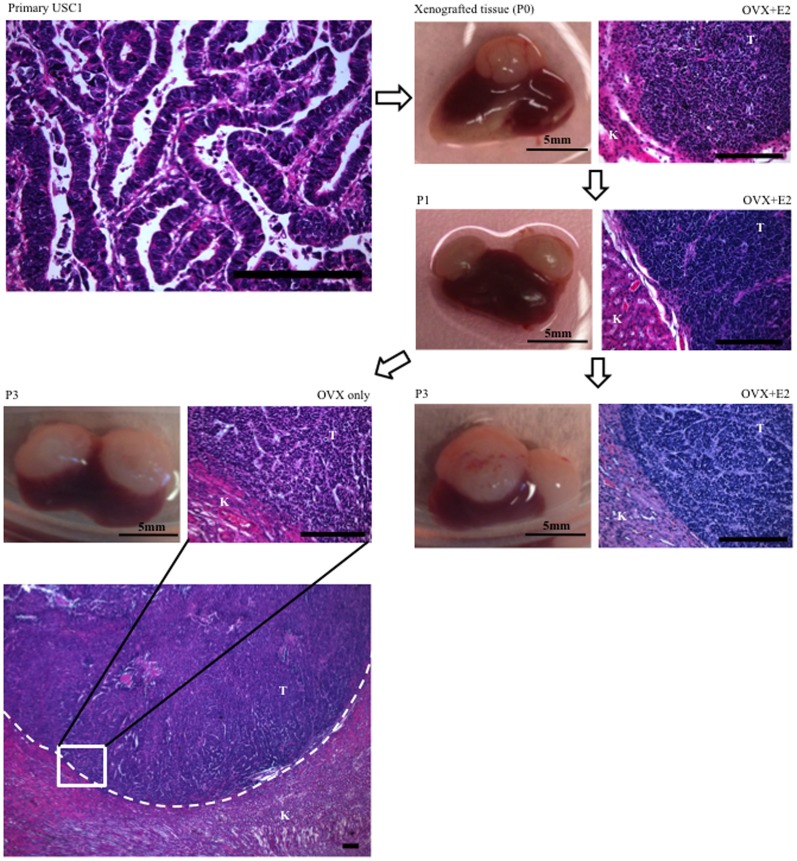
Growth of USC1 under renal capsule of NSG mice. Primary tissues from uterine serous carcinoma (USC1), were transplanted under the renal capsule of immunodefficient ovariectomized (OVX) mice with E2 pellet. At 6 to 8 weeks after transplant, tumor was harvested and cut into multiple 1.5×1.5 mm^3^ pieces, which were grafted under the renal capsule (1–2 pieces/kidney) for continuous passage. At passage 3 (P3), tissues were transplanted in OVX mice with or without E2. USC1 tissue grafts and H&E stainings are shown. T, Tumor; K, Kidney. Scale bar; 200 um.

**Figure 2:**
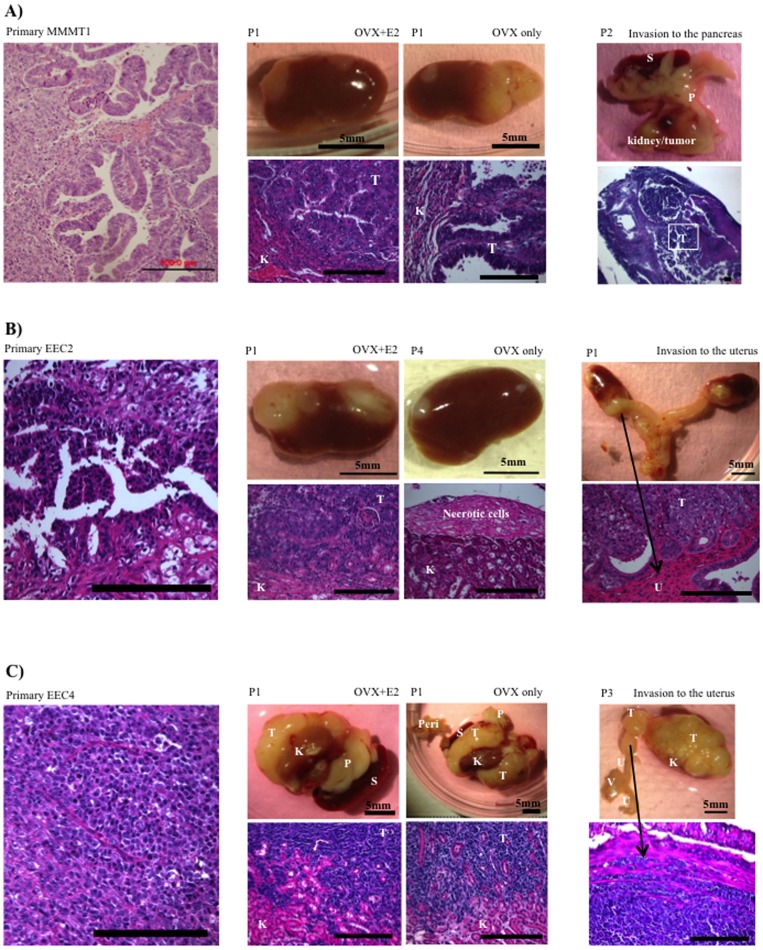
Growth and invasion of MMMT1, EEC2 and EEC4. **A**) Primary tissues from malignant mixed mullerian tumor (MMMT1), were transplanted under the renal capsule of NSG mice that were OVX with or without E2. At 6 to 8 weeks after transplant, tumor was harvested and re-grafted into other mice (1–2 pieces/kidney). At P2, invasion of MMMT1 tissues to pancreas is shown. **B**) Primary tissues from endometrioid adenocarcinoma (EEC2), were transplanted under the renal capsule of OVX mice with E2. At 6 to 8 weeks after transplant, tumor was harvested and re-grafted into other mice (1–2 pieces/kidney). At P4, tissues were transplanted in OVX mice without E2 pellet. At P1, invasion of EEC2 tissues to the uterus is shown. **C**) Primary tissues from endometrioid adenocarcinoma (EEC4), were transplanted under the renal capsule of OVX mice with or without E2. At 6 to 8 weeks after transplant, tumor was harvested and re-grafted into other mice (1–2 pieces/kidney). At P3, invasion of EEC4 tissues to uterus is shown. Left panels show H&E stainings of primary tissues. Middle panels show passaged tissue grafts and H&E stainings. Right panels show invasion of tissue grafts and its H&E stainings. T, Tumor; K, Kidney; S, Spleen; P, Pancreas; Peri, Peritoneum; U, Uterus; V, Vagina. Scale bar; 200 um.

Endometrial tumors were obtained from a total of 11 patients. Four cases of Type II uterine serous carcinoma (USC), 1 case of Type II uterine clear cell carcinoma (UCCC), 1 case of malignant mixed mullerian tumor (MMMT), and 5 cases of endometrioid endometrial carcinoma (EEC) were transplanted under the renal capsule of NSG mice. Among these tumors, USC1, MMMT1, EEC2 and EEC4, established and grew under the renal capsule (Table 1). The engraftment take rates were calculated as the percentage of the number of graphs that grew from the total number of transplanted tissue fragments. USC1 and EEC4 take rates did not differ whether estradiol was present or not in the ovariectomized mice. The engraftment take rate for MMMT1 was higher in the absence of estradiol, while EEC2 had higher take rates with estradiol, demonstrating differential dependence on estrogen for growth. Graphical representation of the xenografts from the 4 cases and corresponding H&E staining are shown in Figs. 1 and 2. Mice harboring the xenografts did not exhibit visible signs of distress during the experimental time period, despite heavy tumor burden in some cases. In addition, mice did not die during the 6–8 weeks of tumor incubation.

**Table 1:** Tumor histopathology.

Case no.	Age of patient	Tumor histopathology
USC1	62	uterine serous carcinoma
MMMT1	49	malignant mixed mullerian tumor
EEC2	52	grade 2 endometrioid adenocarcinoma
EEC4	59	grade 3 endometrioid adenocarcinoma

USC1 was obtained from a patient with a final pathology diagnosis of stage IA grade 3 USC, with lymphovascular space invasion (LVSI). The engraftment take rate was high for this tissue with growth in the majority of grafts (Table 2). Histological examination of the tumor on the kidney revealed no significant invasion into the kidney with a distinct border between the kidney and tumor (Fig. 1). Regardless of whether estradiol was present or not, USC1 tumors grew in a similar manner (Fig. 1, Table 2).

**Table 2:** Engraftment take rates of tumors under the renal capsule.

Case no.	Engraftment take rates^1^	
	OVX^2^+E2	OVX only
**USC1**	**97.1% (34/35)**	**95.2% (20/21)**
**MMMT1**	**41.7% (5/12)**	**78.9% (15/19)**
**EEC2**	**65.7% (23/35)**	**6.3% (1/16)**
**EEC4**	**81.3% (13/16)**	**85.2% (23/27)**

1) ; Engraftment rates are shown by % (a/b).

a; the number of grafts that grew. b; the number of transplanted tissue fragments for all mice.

2) ; OVX, Ovariectomized mice.

MMMT1 from a patient diagnosed with malignant mixed mullerian tumor with LVSI resulted in an engraftment take rate of 42% in the presence of estradiol and 79% without estradiol in the mice (Table 2). In addition, tumors were smaller in mice treated with estradiol compared to no estradiol (Fig. 2A). Visible growth occurred outside the kidney and also infiltrated into the kidney. Remarkably, tumors at second passage showed infiltration into the entire kidney, with local spreading and invasion into the pancreas, which in the mouse is within close proximity to the kidney (Fig. 2A, Table 3). Propagation of P2 tumors in mice with estradiol resulted in suboptimal growth, indicating a negative effect of E2 on growth of MMMT1.

**Table 3:** Characteristics of growing tumors under the renal capsule.

Tissue	E2 requirement	Invasion into kidney	Local invasion to other organs	Local invasion rates (% (a/b))1)	
				OVX+E2	OVX only
USC1	Independent	no	no	0% (0/35)	0% (0/21)
MMMT1	Independent	yes	Pancreas	0% (0/12)	10.5% (2/19)
EEC2	Dependent	yes	Uterus	11.4% (4/35)	0% (0/16)
			Pancreas	2.9% (1/35)	0% (0/16)
EEC4	Independent	yes	Uterus	12.5% (2/16)	7.4% (2/27)
			Spleen	12.5% (2/16)	7.4% (2/27)
			Peritoneum	0% (0/16)	7.4% (2/27)
			Pancreas	18.8% (3/16)	7.4% (2/27)
			**Metastasis**	**Metastatic rates (% (c/d))** 2)	
EEC4			Liver	6.3% (1/16)	0% (0/27)

1) Invasion rates are shown by % (a/b).

a; the number of invaded organs excluding kidneys. b; total number of transplanted tissue fragments (1–2 pieces/kidney).

2) ; Metastatic rates are shown by % (c/d).

c; the number of metastatic lesions at a distal site. d; total number of transplanted tissue fragments.

EEC2 was derived from a patient with stage IA grade 2 endometrioid adenocarcinoma with no LVSI. EEC2 tumors were propagated in OVX mice with E2 implants. To determine E2 dependency, tissues at passage 4 were transplanted in OVX mice without E2. As a result, only 1 tissue out of 16 grew (Table 2). H&E staining showed necrotic areas in the tissue (Fig. 2B). In the presence of estradiol, EEC2 tumors infiltrated the kidney and spread locally to proximal organs including the uterus and pancreas with a local spread ratio of 11.4% and 2.9%, respectively (Fig. 2B, Table 3). Local spread ratio was calculated as the percentage of the number of invaded organs excluding kidneys from the total number of transplanted tissue fragments (1–2 pieces/kidney).

EEC4 originated from a patient with stage IIIC2 grade 3 endometrioid adenocarcinoma with extensive LVSI. This tumor was the most aggressive, with an engraftment take ratio of 81% and 85% with or without estradiol, and significant invasion and local spread to adjacent organs. Tumor was found in the uterus, spleen, liver, peritoneum and pancreas (Fig. 2C, Table 3). In addition, metastatic tumors were observed in the liver (Table 3). No E2 dependency was observed for growth of this tumor as take and spread rates were similar whether E2 was present or not. For this tumor we also assessed whether cryopreserved tumors could be re-propagated. After thawing, tissues were transplanted under the renal capsule in two mice and 7 weeks later, growth of the tumor was observed with similar invasive and spread patterns as the original xenografts.

### Expression of ER, PR, Ki67 and CD31

**Figure 3:**
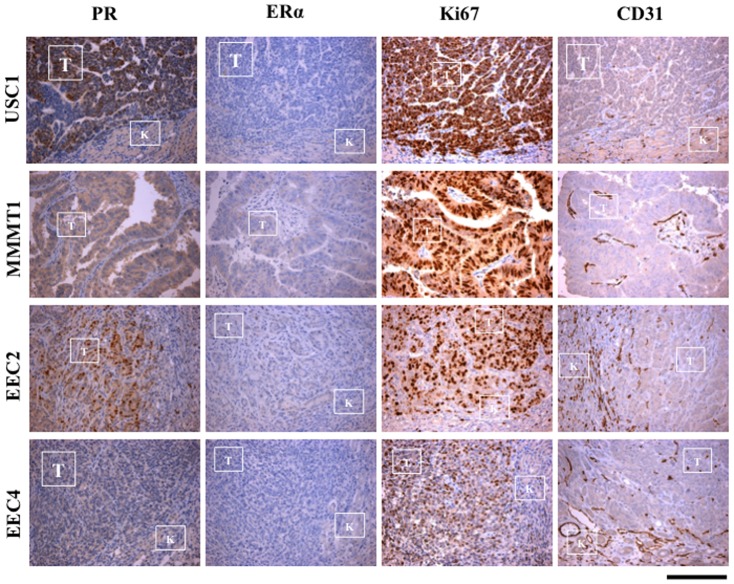
PR, ER, Ki67 and CD31 in xenografted tumors. Immunostaining for PR, ERα, Ki67 and CD31 of USC1, MMMT1, EEC2 and EEC4 xenografted tissues was done. Tumors from OVX mice (no E2) for USC1, MMMT1 and EEC4 and with E2 for EEC2 are shown. USC1, MMMT1, EEC2 and EEC4 tissues were stained at passage 3, 2, 1 and 0, respectivelyT, Tumor; K, Kidney. Scale bar; 200 um.

USC1, MMMT1, EEC2 and EEC4 xenografted tissues, were stained for the steroid hormone receptors, PR and ERα, the proliferation marker, Ki67 and the endothelial cell marker CD31. While USC1 and EEC2 had focal punctate PR staining in specific areas of the tumor, MMMT1 and EEC4 had a more diffuse staining pattern, with EEC4 exhibiting minimal to no staining. In EEC2 xenografts, PR was expressed mostly in the region of the invading front into the kidneys (Fig. 3). ERα levels were low to absent in USC1, MMMT1, EEC2 and EEC4 (Fig. 3). The majority of the cells were positive for Ki67 in USC1, MMMT1, EEC2 and EEC4 tissues, at 6 weeks after transplant indicating active proliferation of tumor cells (Fig. 3). CD31 staining was prevalent within the tumor xenograft in MMMT1, EEC2 and EEC4, especially at the invading front. The level of CD31 staining in USC1 was lower compared to the other tumors (Fig. 3).

### Expression of cytokeratin, vimentin and E-cadherin

**Figure 4:**
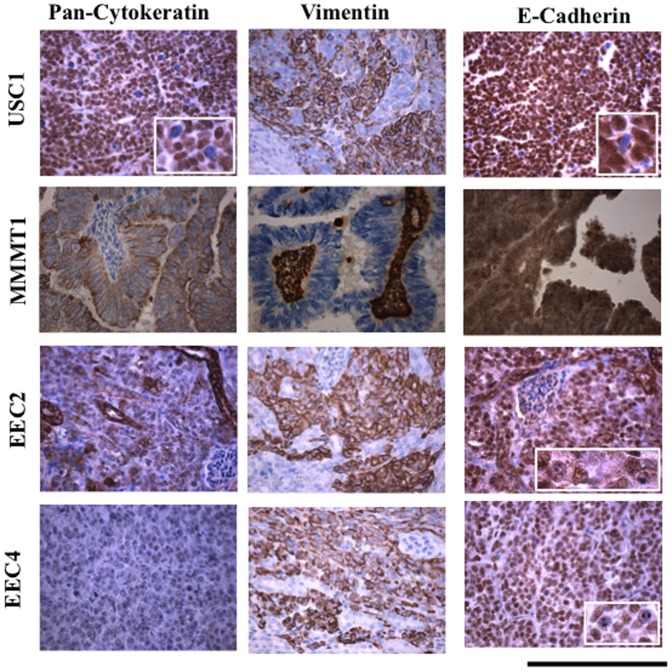
Cytokeratin, Vimentin, and E-cadherin in xenografted tissues. Immunohistochemical staining was done for cytokeratin, vimentin and E-cadherin in xenografted USC1, MMMT1, EEC2 and EEC4 tumors at passage 5, 1, 1 and 0, respectively. Brown color signifies positive staining. Scale bar; 200 um.

Invasion and metastasis of cancer cells is associated with epithelial-mesenchymal transition (EMT) where epithelial cells can transform into motile mesenchymal cells [21]. Tumor xenografts were stained for markers of EMT including cytokeratin, vimentin, and E-cadherin and compared with normal endometrium, hyperplastic endometrium, and grade 1 endometrial cancer (Fig. 4). Glandular epithelium stained strongly positive for cytokeratin in normal endometrium, hyperplasia and grade 1 cancer (Fig. 4). Interestingly, USC1 xenografts stained positive for cytokeratin in the nucleus, while MMMT1 and EEC2 exhibited cytoplasmic staining and EEC4 had very weak to no staining. Both primary and xenografted tissues showed similar patterns of staining with the exception of USC1 where xenografts stained mostly in the nucleus for cytokeratin (Fig. 4, S1 Fig.).

Vimentin was strongly positive in both xenografted and primary tissues for USC1, EEC2 and EEC4 tissues as well as stromal cells in normal endometrium (Fig. 4, S2 Fig.). Grade 1 endometrial cancer and hyperplasia tissues were weakly positive for vimentin in both epithelium and stromal cells (S2 Fig.). The glandular component of MMMT1, showed no staining for vimentin in both xenografted and primary tissues while stroma was positive (Fig. 4, S2 Fig.).

E-cadherin staining in the normal and hyperplastic endometrium and grade 1 tumors localized mainly in the glandular compartment and appeared cytoplasmic (Fig. 4, S3 Fig.). In contrast, nuclear staining of E-cadherin was observed in the xenografts and primary tumors of USC1, EEC2 and EEC4 while MMMT1 exhibited dark cytoplasmic staining. The lack of E-cadherin on the surface of cells would alter the adhesion properties of cells promoting a more motile phenotype.

### Expression of p53 and PTEN

**Figure 5:**
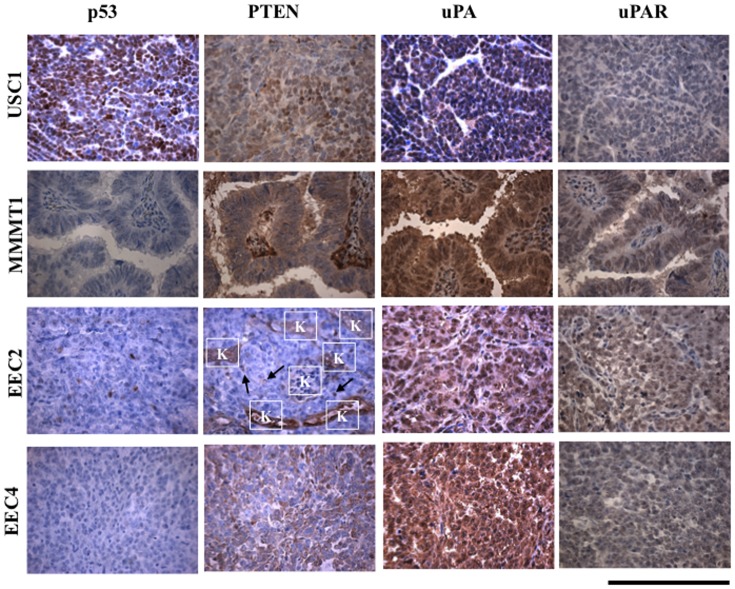
p53, PTEN, uPA, and uPAR in xenografted tissues. Immunohistochemical staining was done for p53, PTEN, uPA and uPAR in xenografted USC1, MMMT1, EEC2 and EEC4 tumors at passage 5, 1, 1 and 0, respectively. Brown color signifies positive staining. Scale bar; 200 um.

The most prevalent genetic alteration in USC occurs in the p53 gene in approximately 90% of cases [3]. The mutation in p53 often manifests as an increase in p53 protein levels [22]. Levels of p53 protein were undetectable in the glands and stroma of normal and hyperplastic endometrium and grade 1 endometrial cancer (S4 Fig.). Similarly, p53 staining was minimal to absent in MMMT1, EEC2 and EEC4 in both the xenografted and primary tumors (Fig. 5, S4 Fig.). In contrast, p53 staining was strongly positive in USC1 xenografted and primary tumors. Type I EEC is characterized by a variety of genetic alterations with the most prevalent mutations in the PTEN gene [3]. All xenografted and primary tumors stained for PTEN with the exception of EEC2 which showed minimal to no staining in the tumor cells (Fig. 5, S5 Fig.). Comparatively, the glandular compartment of hyperplasia and grade 1 endometrial cancer showed no staining of PTEN whereas normal endometrium stained for PTEN in the glands and stroma (S5 Fig.).

### Expression of Urokinase plasminogen activator (UPA) system

UPA and its receptor UPAR promotes proteolysis that enhances tumor growth and invasion. Both UPA and UPAR have been shown to be expressed in advanced cancers [23]. All tumor xenografts and primary tumors stained positively for UPA (Fig. 5, S6 Fig.). Similarly, normal and hyperplastic endometrium and grade 1 endometrial cancer, stained for UPA in both the glandular and stromal compartments (S6 Fig.). In contrast, staining for UPAR was evident in the invasive tumors, MMMT1, EEC2 and EEC4 with little staining was observed in USC1 (Fig. 5). UPAR levels were absent in grade 1 endometrial cancer and normal endometrium (S7 Fig.). These results suggest that expression of UPAR may contribute to the invasive nature of the endometrial tumors in our system.

## Discussion

The purpose of this study was to establish patient derived tumor xenografts of primary endometrial cancer tissues for continued propagation to provide a model to study invasion and metastasis. We report here establishment of tumors from four patients of different endometrial cancer types and grades that show differential invasive and metastatic capacity. The xenografts retain characteristics of the original tumor and display features that are unique to Type 1 or Type II endometrial cancer.

As endometrial tumors become more aggressive and poorly differentiated, expression of hormone receptors, ER and PR diminishes, and their hormone responsiveness changes. The dependence of tumor grafts to E2 was demonstrated here. USC1, MMMT1, and EEC4 did not require E2 for grafts to grow. This may be due to the low levels of ER in the tumors. In contrast, EEC2 maintained E2 dependency despite the low levels of ER detected. E2 could be a ligand to the G-protein coupled receptor 30 (GPR30), which is overexpressed in high grade endometrial cancer [24]. Additionally, it is possible that GPR30 mediates non-transcriptional effects of estrogen on the activation of PI3K/Akt pathway in this tumor, and promote growth [25]. Interestingly, while all four xenografted tissues were ERα low to negative, all grafted tumors expressed varying levels of PR. The PR expressing cells of EEC2 were localized around the invading front of the kidney. The reason for expression of PR in this particular area and its role in invasion remains unclear. In fact, the mechanism of action of progesterone through its receptor in advanced, invasive endometrial carcinoma is unknown. Genes that are regulated by PR in the normal endometrium are different than those in endometrial cancer [26]. Given the pleiotropic activity of PR which is dependent on the cellular environment [15], it is possible that PR could have both growth-, invasion- and metastasis- promoting or inhibiting actions on the tumor cell. Our patient xenograft model would be a useful tool to decipher the role of progesterone on these tumors.

In order for cancer cells to invade, cell-cell adhesion must be lost to obtain cell motility and break away from the tumor tissue. EMT is involved in the dissemination of individual carcinoma cells from primary carcinoma tissues, either transiently or stably [27], [28]. Loss of E-cadherin is the initial step of EMT, permitting invasion and metastasis in many carcinomas. In USC1, EEC2 and EEC4, E-cadherin was localized to the nucleus whereas MMMT1, grade 1 endometrial cancer, hyperplasia, and normal endometrium exhibited dark cytoplasmic staining. Studies have shown nuclear localization of E-cadherin in both benign and malignant tumors [29], [30]. Cleaved fragments of E-cadherin have been reported to translocate to the nucleus [31]. Loss of intact transmembrane E-cadherin would inevitably decrease cell to cell adhesion.

The urokinase plasminogen activator (UPA) system can cause degradation of extracellular matrix, enhance angiogenesis and lead to invasion and metastasis [32]. There is little known about the UPA system in endometrial cancer. Previously we identified increased UPA mRNA expression in a uterine serous carcinoma cell line (SPEC2) compared to the low grade endometrioid carcinoma cell line (Ishikawa) [33]. The receptor UPAR has been shown to be present at higher levels in patients with aggressive and late stage endometrial cancers [32], [34]. In our study, UPA staining was observed in all the tumors tested whereas UPAR was expressed in the tumors that invaded through the kidney and local organs suggesting that UPAR could be targeted to inhibit invasion in advanced endometrial cancer.

In summary, we have successfully established and propagated patient derived endometrial tumors from 4 cases using the renal capsule xenograft system. This model could be used to test novel compounds as well as combination therapies and is superior to the conventional cell line xenograft models. In addition, the biology of the tumor can easily be assessed to identify predictive markers for responses to treatment regimens which are currently lacking for advanced and recurrent endometrial cancer. Despite the good prognosis that is associated with low grade endometrial cancer, especially when detected early, the advanced cases are lethal with very little to no effective treatments for this disease. Studying patient tumors as xenografts will provide the much needed information to improve on therapies for aggressive endometrial cancer.

## Supporting Information

S1 Fig **Cytokeratin in primary and xenografted tissues.** Immunohistochemical staining was done for vimentin in primary and xenografted USC1, MMMT1, EEC2 and EEC4 tumors at passage 5, 1, 1 and 0, respectively. Staining was done for normal and hyperplastic endometrium and grade 1 endometrial cancer tissues. Brown color signifies positive staining. Scale bar; 200 um.
(TIFF)

S2 Fig **Vimentin in primary and xenografted tissues.** Immunohistochemical staining was done for vimentin in primary and xenografted USC1, MMMT1, EEC2 and EEC4 tumors at passage 5, 1, 1 and 0, respectively. Staining was done for normal and hyperplastic endometrium and grade 1 endometrial cancer tissues. Brown color signifies positive staining. Scale bar; 200 um.
(TIFF)

S3 Fig **E-cadherin in primary and xenografted tissues.** Immunohistochemical staining was done for E-cadherin in primary and xenografted USC1, MMMT1, EEC2 and EEC4 tumors at passage 5, 1, 1 and 0, respectively. Staining was done for normal and hyperplastic endometrium and grade 1 endometrial cancer tissues. Brown color signifies positive staining. Scale bar; 200 um.
(TIFF)

S4 Fig **p53 in primary and xenografted tissues.** Immunohistochemical staining was done for p53 in primary and xenografted USC1, MMMT1, EEC2 and EEC4 tumors at passage 5, 1, 1 and 0, respectively. Staining was done for normal and hyperplastic endometrium and grade 1 endometrial cancer tissues. Brown color signifies positive staining. Scale bar; 200 um.
(TIFF)

S5 Fig **PTEN in primary and xenografted tissues.** Immunohistochemical staining was done for PTEN in primary and xenografted USC1, MMMT1, EEC2 and EEC4 tumors at passage 5, 1, 1 and 0, respectively. Staining was done for normal and hyperplastic endometrium and grade 1 endometrial cancer tissues. Arrows show PTEN positive cells in EEC2. K, Kidney; Brown color signifies positive staining. Scale bar; 200 um.
(TIFF)

S6 Fig **UPA in primary and xenografted tissues.** Immunohistochemical staining was done for UPA in primary and xenografted USC1, MMMT1, EEC2 and EEC4 tumors at passage 3, 1, 1 and 0, respectively. Staining was done for normal and hyperplastic endometrium and grade 1 endometrial cancer tissues. Brown color signifies positive staining. Scale bar; 200 um.
(TIFF)

S7 Fig **UPAR levels in primary and xenografted tissues.** Immunohistochemical staining was done for UPAR in primary and xenografted USC1, MMMT1, EEC2 and EEC4 tumors at passage 5, 1, 1 and 0, respectively. Staining was done for normal endometrium and grade 1 endometrial cancer tissues. Brown color signifies positive staining. Scale bar; 200 um.
(TIFF)

S8 Fig **ARRIVE checklist.**
(PDF)
