# Supplementary figures and images for: Establishment of Human Patient-Derived Endometrial Cancer Xenografts in NOD scid Gamma Mice for the Study of Invasion and Metastasis
Source: PLoS One. 2014 Dec 26;9(12):e116064. doi: 10.1371/journal.pone.0116064 (PMC4277433; doi:10.1371/journal.pone.0116064)

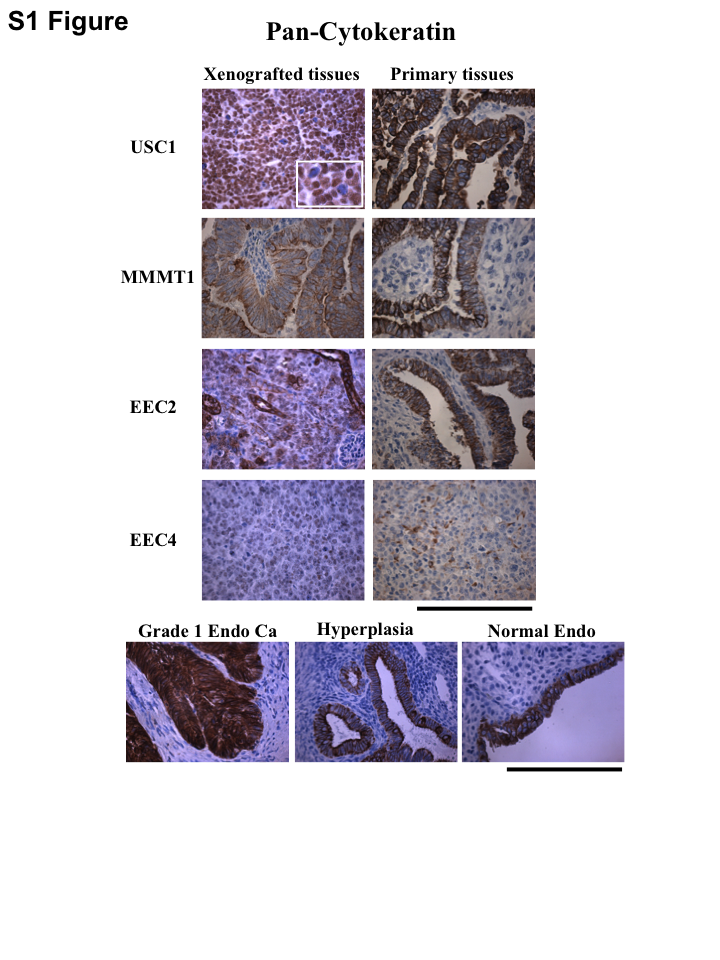

Supplement: S1 Fig — Cytokeratin in primary and xenografted tissues. Immunohistochemical staining was done for vimentin in primary and xenografted USC1, MMMT1, EEC2 and EEC4 tumors at passage 5, 1, 1 and 0, respectively. Staining was done for normal and hyperplastic endometrium and grade 1 endometrial cancer tissues. Brown color signifies positive staining. Scale bar; 200 um. (TIFF) [file pone.0116064.s001.tiff]

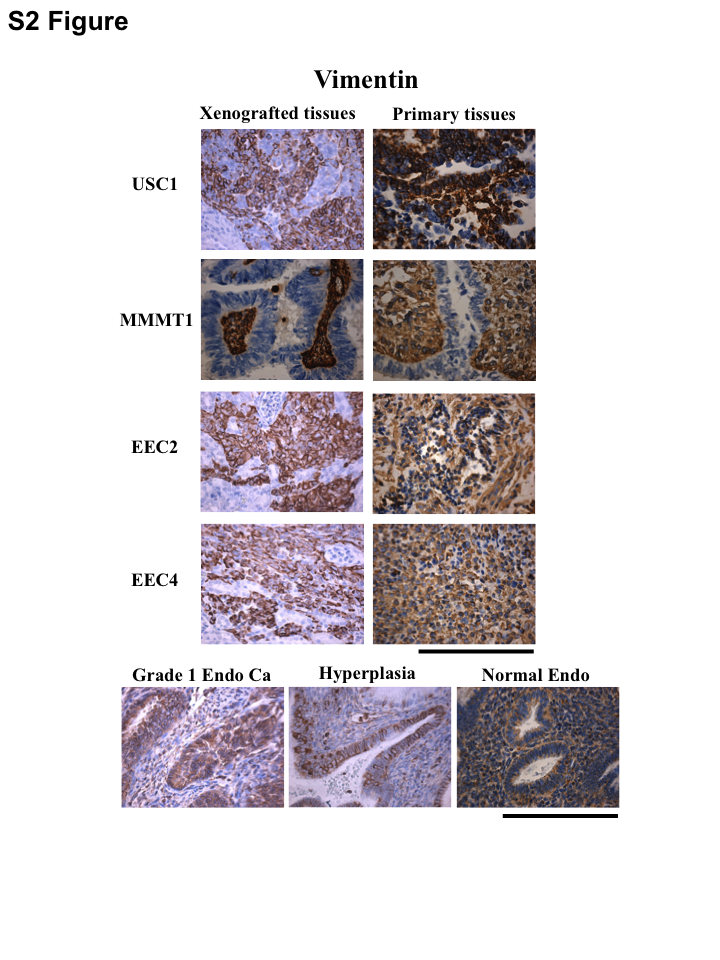

Supplement: S2 Fig — Vimentin in primary and xenografted tissues. Immunohistochemical staining was done for vimentin in primary and xenografted USC1, MMMT1, EEC2 and EEC4 tumors at passage 5, 1, 1 and 0, respectively. Staining was done for normal and hyperplastic endometrium and grade 1 endometrial cancer tissues. Brown color signifies positive staining. Scale bar; 200 um. (TIFF) [file pone.0116064.s002.tiff]

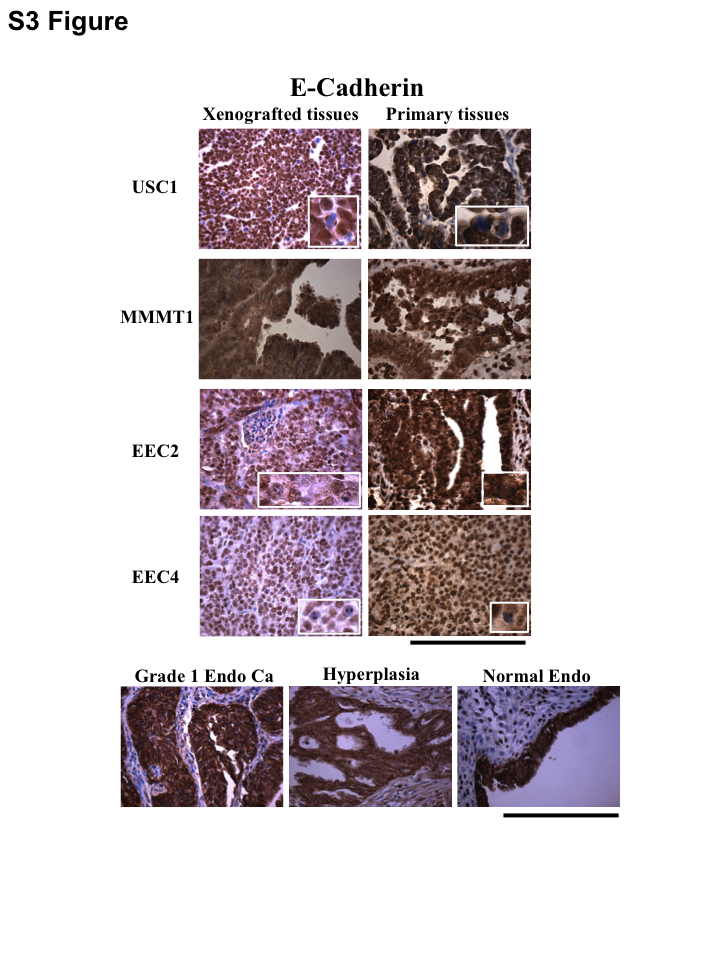

Supplement: S3 Fig — E-cadherin in primary and xenografted tissues. Immunohistochemical staining was done for E-cadherin in primary and xenografted USC1, MMMT1, EEC2 and EEC4 tumors at passage 5, 1, 1 and 0, respectively. Staining was done for normal and hyperplastic endometrium and grade 1 endometrial cancer tissues. Brown color signifies positive staining. Scale bar; 200 um. (TIFF) [file pone.0116064.s003.tiff]

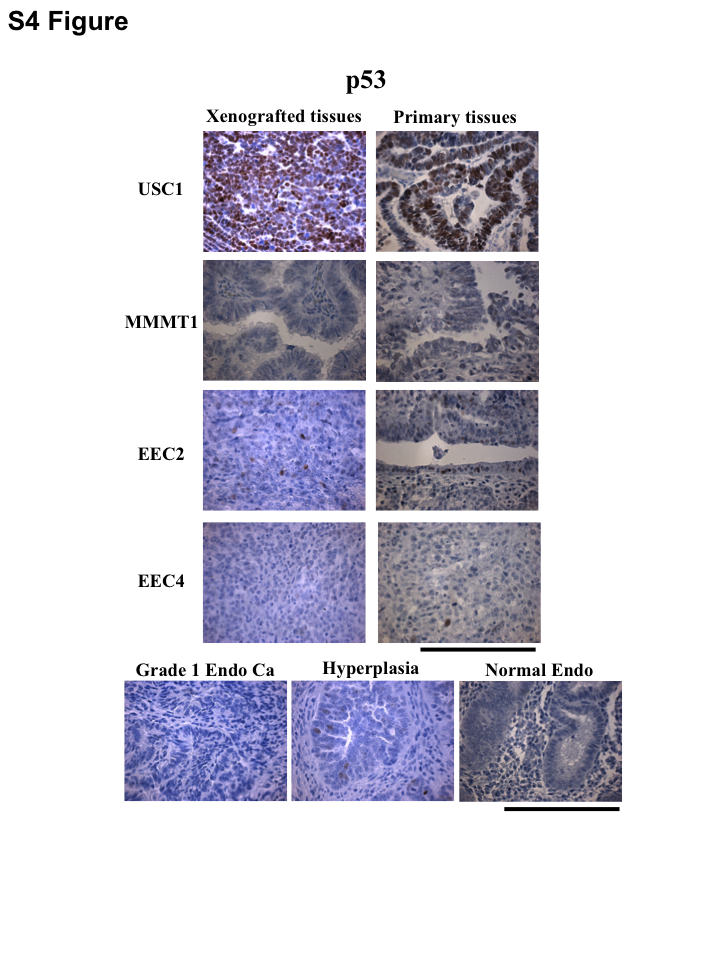

Supplement: S4 Fig — p53 in primary and xenografted tissues. Immunohistochemical staining was done for p53 in primary and xenografted USC1, MMMT1, EEC2 and EEC4 tumors at passage 5, 1, 1 and 0, respectively. Staining was done for normal and hyperplastic endometrium and grade 1 endometrial cancer tissues. Brown color signifies positive staining. Scale bar; 200 um. (TIFF) [file pone.0116064.s004.tiff]

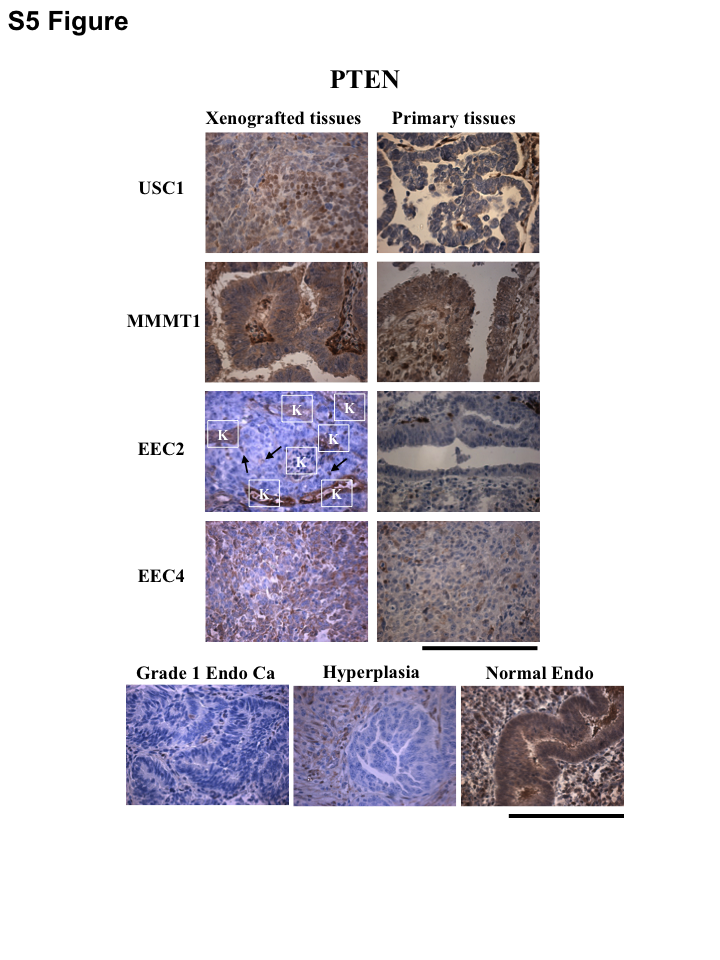

Supplement: S5 Fig — PTEN in primary and xenografted tissues. Immunohistochemical staining was done for PTEN in primary and xenografted USC1, MMMT1, EEC2 and EEC4 tumors at passage 5, 1, 1 and 0, respectively. Staining was done for normal and hyperplastic endometrium and grade 1 endometrial cancer tissues. Arrows show PTEN positive cells in EEC2. K, Kidney; Brown color signifies positive staining. Scale bar; 200 um. (TIFF) [file pone.0116064.s005.tiff]

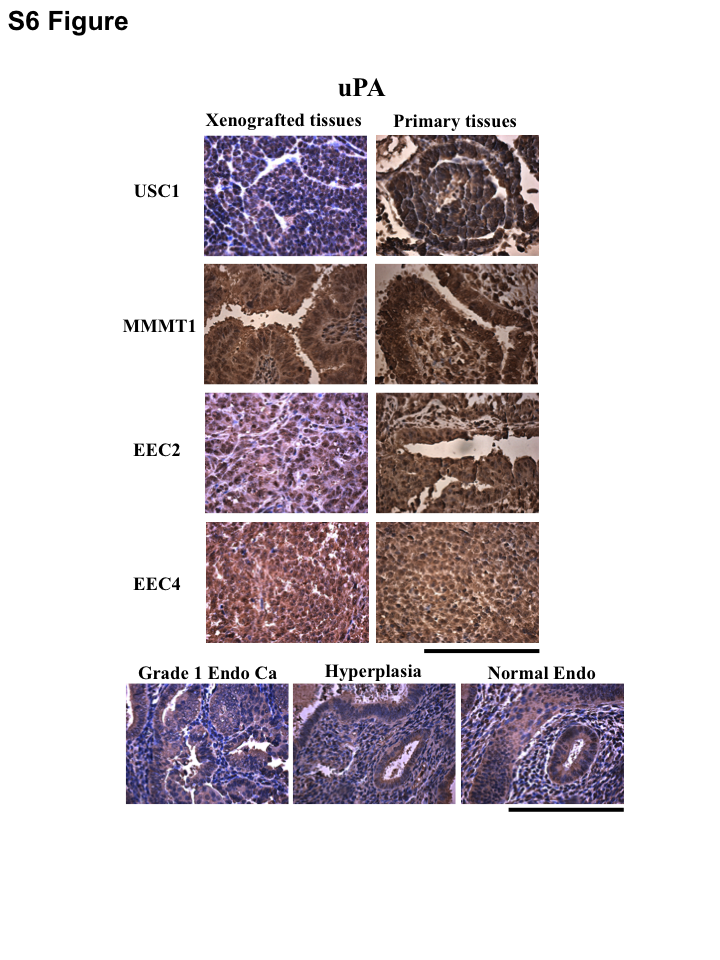

Supplement: S6 Fig — UPA in primary and xenografted tissues. Immunohistochemical staining was done for UPA in primary and xenografted USC1, MMMT1, EEC2 and EEC4 tumors at passage 3, 1, 1 and 0, respectively. Staining was done for normal and hyperplastic endometrium and grade 1 endometrial cancer tissues. Brown color signifies positive staining. Scale bar; 200 um. (TIFF) [file pone.0116064.s006.tiff]

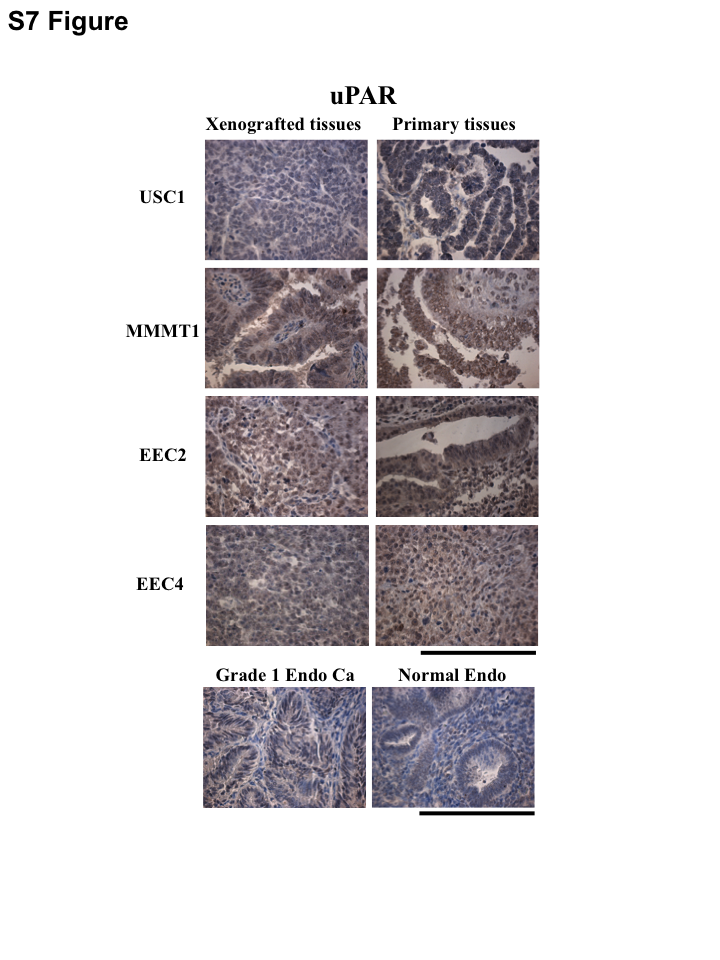

Supplement: S7 Fig — UPAR levels in primary and xenografted tissues. Immunohistochemical staining was done for UPAR in primary and xenografted USC1, MMMT1, EEC2 and EEC4 tumors at passage 5, 1, 1 and 0, respectively. Staining was done for normal endometrium and grade 1 endometrial cancer tissues. Brown color signifies positive staining. Scale bar; 200 um. (TIFF) [file pone.0116064.s007.tiff]
